# Supplementary material for: Identification of human genetic variants controlling circular RNA expression
Source: RNA. 2019 Dec;25(12):1765–78. doi: 10.1261/rna.071654.119 (PMC6859849; doi:10.1261/rna.071654.119)
Supplement: Supplemental Material [file supp_25_12_1765__index.html]

Identification of human genetic variants controlling circular RNA expression — Supplemental Material 

# Identification of human genetic variants controlling circular RNA expression

## Supplemental Material

- Supplemental\_Figure\_1.pdf
- Supplemental\_Figure\_2.pdf
- Supplemental\_Figure\_3.pdf
- Supplemental\_Figure\_4.pdf
- Supplemental\_Figure\_5.pdf
- Supplemental\_File\_S1.xlsx
- Supplemental\_File\_S2.xlsx
- Supplemental\_File\_S3.xlsx
- Supplemental\_File\_S4.xlsx
