# Supplementary material for: Identification of human genetic variants controlling circular RNA expression
Source: RNA. 2019 Dec;25(12):1765–78. doi: 10.1261/rna.071654.119 (PMC6859849; doi:10.1261/rna.071654.119)
Supplement: Supplemental Material [file supp_071654.119_Supplemental_Figure_2.pdf]

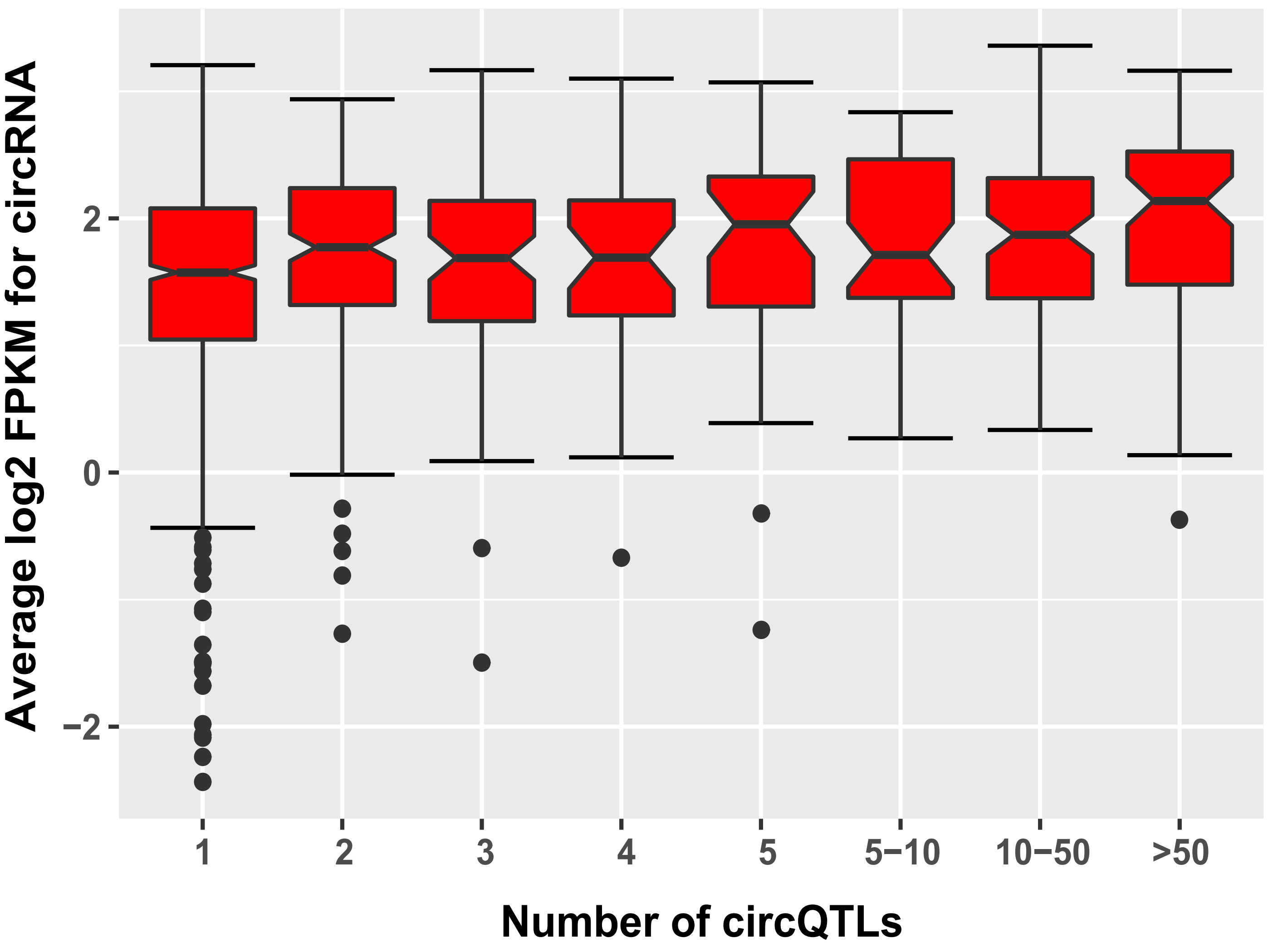

Supplementary Figure 2: Relative Expression of circRNAs vs number of circQTLs detected.
Boxplots of Average circRNA expression from 358 EUR samples.
The data is categorized as per the number of circQTLs identified for each gene.
