# Supplementary material for: Identification of human genetic variants controlling circular RNA expression
Source: RNA. 2019 Dec;25(12):1765–78. doi: 10.1261/rna.071654.119 (PMC6859849; doi:10.1261/rna.071654.119)
Supplement: Supplemental Material [file supp_071654.119_Supplemental_Figure_3.pdf]

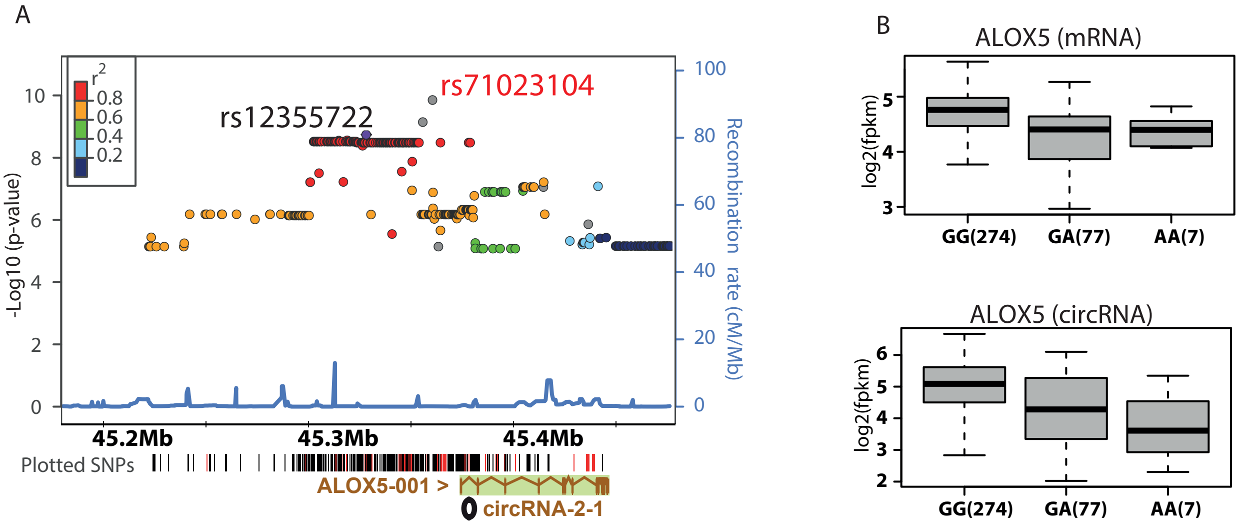

Supplementary Figure 3: Region association plot for ALOX5 circRNA. (A)The plot is centered at the locus rs12355722 (purple diamond), which is associated with both circRNA and mRNA expression of ALOX5 gene. The circQTL only SNP (rs71023104) shows highest association with ALOX5 circRNA processed from its exons 1 and 2. In the plotted SNPs, red color indicates variants associated with only circRNA expression and black shows variants associated with both circRNA and mRNA expression. The values of r2 are based on the CEU HapMap 2 samples. The CEU HapMap 2 recombination rates are indicated in blue on the right y-axes as obtained using LocusZoom (http://csg.sph.umich.edu/locuszoom). (B) The circQTL and eQTL variant rs12355722 is associated with both circRNA and mRNA expression of the gene and shows a higher effect size beta (circRNA:mRNA -0.47:-0.35) for circRNA. Number of samples in each genotype group is shown in the brackets.
