# Supplementary material for: Identification of human genetic variants controlling circular RNA expression
Source: RNA. 2019 Dec;25(12):1765–78. doi: 10.1261/rna.071654.119 (PMC6859849; doi:10.1261/rna.071654.119)
Supplement: Supplemental Material [file supp_071654.119_Supplemental_Figure_5.pdf]

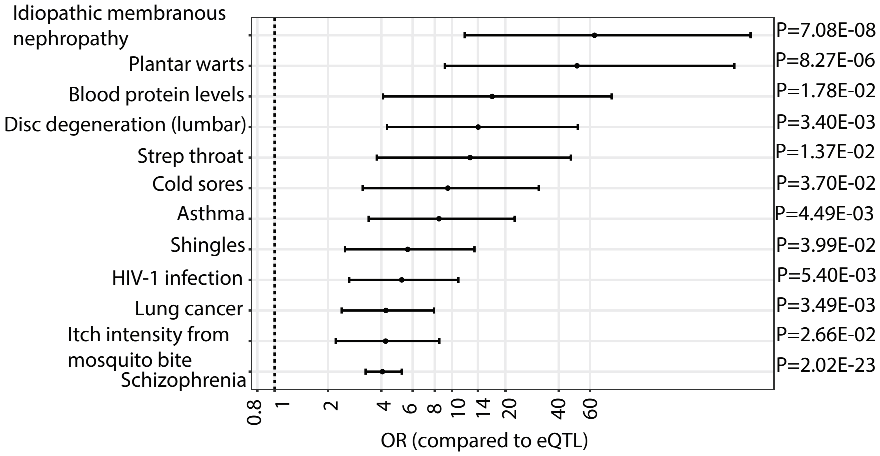

Supplementary Figure 5. Enrichment of circQTL SNPs among disease associated loci. Results of enrichment analysis of circQTL SNPs associated with various traits in GWAS catalog [39] and arranged by odds ratio. A total of 306 traits were tested and only 12 were found to show statistically significant enrichments. The nominal P-values obtained from one-tailed Fisher's exact test were Bonferroni corrected for the total number of 306 traits tested and a p-value cut-off of 1.6E-04 (0.05/306) was used as a significance threshold. Bars indicate 95% confidence intervals.
